# Dr. Goodell’s Mixture of the Four Chlorides

**Published:** 1883-10

**Authors:** 


### Dr. Goodell's Mixture of the Four Chlorides.

The following is known as Dr. Goodell's mixture of the "four chlorides," which he prescribes as an alterative tonic:

| | | |
|---|---|---|
| ℞. | Hydrarg. bichlor, . . . . . | grains j-ij. |
| | Liq. arsen. chlor, . . . . . | drachm j. |
| | Acidi. hydrochlor, dil. | |
| | Tr. ferri. chlor., of each, . . . | drachms ij. |
| | Syr. Zingib, . . . . . . | ounces ij. |
| | Aquæ ad. . . . . . | ounces vj. |

M. Sig.—two teaspoonfuls three times daily in water, after meals.—*Canada Lancet.*

